# Diversity in radiology: the right thing to do, the smart thing to do

**DOI:** 10.1007/s00247-022-05416-5

**Published:** 2022-06-23

**Authors:** Theodore R. Hall, Kathleen Brown

**Affiliations:** 1 UCLA Department of Radiology, Section of Pediatric Imaging, 757 Westwood Plaza, Los Angeles, CA 90095 USA; 2 UCLA Department of Radiology, Section of Cardiothoracic Imaging, Los Angeles, CA USA

**Keywords:** Amplification cascade, Diversity, Radiology, Unconscious bias

## Abstract

In the 10-year period between the last two U.S. population censuses there have been significant changes in the U.S. population demography. The changes in self-reporting of racial and ethnic identity afforded by the most recent U.S. population census in 2020 have given citizens the opportunity to be represented in ways that truly reflect how they wish to be identified. At the same time, the diversity of the health care workforce in radiology has not reflected a similar change. While there have been small incremental changes for underrepresented groups (African Americans/Blacks, Hispanic ethnicity, and the group American Indian/Alaska Native/Native Hawaiian/Pacific Islander), these changes have not kept pace with the ever-changing demographics of the U.S. population. Part of the answer for these very modest gains must lie with our selection processes for identifying potential candidates from underrepresented in medicine groups (URiM) for acceptance to our medical schools, residency programs and employment opportunities as practicing physicians and faculty members. While the strategies employed have had some measure of success, our best efforts to increase diversity in our specialty, and in medicine in general, are being undermined by our biases and our traditional methods for identifying talents.

## Introduction

On Jan. 20, 2020, the Centers for Disease Control (CDC) reported the first U.S.-laboratory-confirmed case of coronavirus disease 2019 (COVID-19) in the United States from samples taken on Jan. 18 in Washington State. Over the next several months the collision of medicine and a worldwide social justice movement brought attention to many of the inequities that have long existed in our society. News outlets all over the nation reported on the disproportionate effect of COVID-19 on communities of color and workers in service industries, and unconscionable health care disparities. Virtually every state in the union posted data that demonstrated a disproportionate number of cases and deaths among marginalized segments of the population, in particular African Americans. This is the background of our state of affairs that has prompted so much interest and discussion about diversity in medicine and, in particular, radiology.

## The 2020 United States population census

The U.S. Census projections demonstrate the diverse future of the country. The U.S. population grew in the 10-year period between the 2010 and 2020 censuses by 7.4%, from 308,745,538 to 331,893,745 [1]. Differences in reporting race from 2010 to 2020 were a result of expert research and the findings of the 2015 National Content Test about the impacts of question format on race and ethnicity reporting [2]. This resulted in the addition of self-identification categories that included “some other race” alone or in combination with other populations, and the “multiracial” population. The discussion of race is now framed using the concepts of race alone, race in combination, and race alone or in combination, because these three concepts are essential to understanding how the American people self-identify and the changing demographics of the country [3].

According to the 2020 Census, the White population remained the largest race or ethnicity group in the United States, with 204.3 million people identifying as White alone. Overall, 235.4 million people reported White alone or in combination with another group. However, the White alone population decreased by 8.6% in the 10-year period between the two most recent censuses [3].

For Black or African American in combination, there was an 88.7% increase in population in this same 10-year period. The population of Black or African American alone or in combination with another race comprised 14.2% of the total population in the 2020 Census, compared to 12.6% in 2010 [3].

Between 2010 and 2020, the percentage of the population identifying as Hispanic or Latino increased by 2.4%, from 16.3% to 18.7% of the total population. The growth of this ethnic group accounts for more than half of the U.S. population growth in the period between the last two censuses. The number of people of Hispanic or Latino origin reporting more than one race increased 567%. As a result, the Hispanic or Latino population grew by 23% between the 2010 and 2020 censuses. The increase is largely a result of the question redesign, with the two separate questions for race and ethnicity allowing for a more thorough and accurate depiction of how people wish to self-describe [3].

The American Indian and Alaska Native in combination population grew by 160%. This growth represents an additional 5.9 million people who self-identified as American Indian and Alaska Native and another race group in 2020, such as White or Black or African American. The population of American Indian and Alaska Native alone or in combination comprised 2.9% of the total population in 2020, up from 1.7% in 2010 [3]. The Asian population grew by 35.5% between 2010 and 2020, comprising 6% of all respondents who identified as Asian alone. The Asian in combination population increased by 55.5% [3].

According to data analysis for the 2020 Census, more than half of those who self-identified as Native Hawaiian and other Pacific Islander identified with more than one race. There was no significant change in the percentage of the population who identified as Native Hawaiian and other Pacific Islander alone between the 2010 and 2020 censuses, representing 0.2% of the population. However, there was a significant change in growth of the Native Hawaiian and other Pacific Islander in combination — 30.8% — compared to the Native Hawaiian and other Pacific Islander alone population at 27.8% [3].

The multiracial population has changed considerably since 2010. It was measured at 9 million people in 2010 and 33.8 million people in 2020, a 276% increase. Those who self-identified as multiracial in the 2020 Census represent 10.2% of the total population. The change is largely a result of how people prefer to self-identify [3].

The “some other race” category was the second-largest alone or in combination, representing 15.1% of the total population [3].

With the exception of the White-alone population, all racial and ethnic groups increased in the 2020 U.S. Census, with the most significant shifts occurring in the number of people of Hispanic or Latino origin reporting more than one race and in the number of people who self-identified as American Indian and Alaska Native and another racial group, such as White or Black or African American. It is clear from the data analysis of the 2020 Census that there have been significant demographic shifts from 2010 and these shifts reflect a greater understanding of how people view their self-identification as more than Black and White. This has important implications for leadership in medicine when attempting to address the diversity of our health care workforce.

## Underrepresented in medicine (URiM) representation

The Association of American Medical Colleges (AAMC) defines the term “underrepresented in medicine” — URiM — to include racial and ethnic populations that are underrepresented in the medical profession relative to their numbers in the general population. In the vast majority of cases, this includes African Americans/Blacks, people of Hispanic ethnicity and the group American Indian/Alaska Native/Native Hawaiian/Pacific Islander. Because of regional differences in populations, some Asian populations might also be considered in the URiM category.

The URiM groups comprised 30% of the population in the 2010 U.S. Census and 33.4% of the population in the 2020 Census [4]. Among the top 20 largest U.S. medical specialty training programs in 2012, 13.8% of trainees were URiMs. In terms of minority representation, radiology ranked 16th for American Indian/Alaska Native/Hawaiian Native/Pacific Islander, 18th for Black, 19th for Hispanic ethnicity and 18th for all URiM trainees [4].

In a comprehensive review of diversity of the radiology physician workforce by race, Hispanic ethnicity and gender in the context of an available pipeline of medical students, Chapman et al. [5] used publicly available registries from the American Medical Association, AAMC and U.S. Census to assess differences between (1) diagnostic radiology residents, practicing physicians, academic faculty members, subspecialty trainees, residency applicants and medical school graduates and (2) the U.S. population. Their research showed that individuals who self-identified as Black; Hispanic or Latino; or the group American Indian/Alaska Native, Native Hawaiian or Pacific Islander were significantly underrepresented when diagnostic radiology residents were compared with medical school graduates, and when practicing diagnostic radiology physicians and faculty were compared to the 2010 U.S. Census numbers by individual groups [5].

While Blacks comprised 12.6% of the U.S. population in the 2010 Census, they remained underrepresented among medical school graduates at 6.8%, and diagnostic radiology residency applicants and diagnostic radiology residents at 5.6% and 3.1%, respectively, with continued underrepresentation among practicing diagnostic radiologists and academic faculty members [3, 5]. American Indians, Alaska Natives, Native Hawaiians and Pacific Islanders comprised 1.1% of the U.S. population in the 2010 Census. Their numbers are disproportionately underrepresented by all comparisons for medical student graduates, diagnostic radiology residency applicants, diagnostic radiology residents, practicing diagnostic radiologists and faculty members [3, 5]. For those who identify as Hispanic (Latinx), the proportion of medical school graduates (7.4%) was significantly different compared to the group’s percentage of the U.S. population [3, 5].

Although Asian Americans comprised 4.8% of the U.S. population in the 2010 U.S. Census, they were disproportionately over-represented among medical school graduates (20.8%), diagnostic radiology applicants (24.4%), practicing diagnostic radiologists (13.4%) and faculty members (9.2%) [5].

Based on the 2020 U.S population census and a comparison of the U.S. population with U.S. medical school graduates, radiology applicants, residents, practicing physicians and faculty members, there have been modest gains over time. URiM graduates from U.S. medical schools comprised 12% (2,348 of the 19,646 graduates) — a 21.5% decrease from the 15.3% (2,572 of the 16,835) URiM graduates in 2010. Women comprised 48.3% of medical school graduates in 2010, versus 49.5% and 50.5% in 2020 and 2021, respectively [6].

Although the number of Hispanic medical school graduates decreased by 2.1% in the 10 years between 2010 and 2020, radiology made modest gains in applicants (5.9% to 8.1%), residents (4.8% to 6.9%) and practicing physicians (3.8% to 4.4%); there was a decrease in Hispanic or Latino academic radiologists from 4.3% to 2.5% [7–9] (Table 1; [5]). An analysis of Black representation in radiology showed smaller gains for applicants (5.6% to 6.3%), residents (3.1% to 3.9%), practicing physicians (2.1% to 2.6%) and academic radiologists (2.0% to 2.3%). For the American Indian/Alaska Native/Native Hawaiian/Pacific Islander group, there were decreases in applicants (1.7% to 1.0%) and residents (0.4% to 0.2%), with slight increases in both practicing physicians (0.1% to 0.3%) and academic radiologists (0.1% to 0.2%) [7–9] (Table 1). As discussed, changes in the questions regarding race and ethnicity might have enhanced definitions of self-identification in the 2020 U.S. Census and these enhancements might account for small changes in percentages of demographic representation of the racial and ethnic groups.

**Table 1:** 2020 demographic distribution by gender, Hispanic ethnicity and race of the U.S. population, U.S. medical school graduates, diagnostic radiology applicants, residents, practicing physicians and faculty members

Group	Men	Women	Hispanic	Non-Hispanic	White	Asian	Black	AI/AN/NH/PI	Multi-race	Total Non-duplicated
2020 U.S. Census	163.2 M (49.2)	168.6 M (50.8)	**60.7 M (18.5)**	197.2 M (60.1)	250.4 M (76.3)	**19.4 M (5.9)**	**44.0 M (13.4)**	**4.9 M (1.5)**	9.2 M (2.8)	328,239,523
U.S. medical school graduates	10,280 (50.4)	10,110 (49.5)	*1,063 (5.3)*	504 (2.5)	**10,897 (54.6)**	**4,299 (21.6)**	*1,238 (6.2)*	*47 (0.3)*	1,598 (8.0)	19,646
Diagnostic radiology applicants^a^	1,743 (72.5)	662 *(27.5)*	**171 (8.1)**	254 (12.1)	**986 (47.1)**	528 (25.2)	**132 (6.3)**	21 (1.0)		2,092
Diagnostic radiology residents^b^	3,459 *(64.1)*	**1,244 (35.9)**	**302 (6.9)**	222 (5.0)	*2,583 (59.2)*	**1,190 (27.8)**	**173 (3.9)**	*8 (0.2)*	187 (4.3)	4,362
Diagnostic radiology practicing physicians	20,595 (73.5)	**7,413 (26.5)**	**1,106 (4.4)**	480 (1.9)	1,8,252 (73.0)	4,240 (16.9)	**658 (2.6)**	**75 (0.3)**	187 (0.7)	**28,008**
Diagnostic radiology faculty^c^	7,161 (70.1)	**3,052 (29.9)**	*239 (2.5)*	216 (2.2)	*5,915 (62.0)*	2,718 (28.5)	**228 (2.3)**	**21 (0.2)**	198 (2.0)	10,213

Based on comparison of the 2010 US population census and the recently completed 2020 U.S population census numbers in **bold** represent increases, *italics* represent declines with percentages for both in parentheses for the U.S. population, U.S. medical school graduates, radiology applicants, residents, practicing physicians, and faculty (Comparison of data abstracted from [5])

*AI/AN/NH/PI* American Indian/Alaskan native/Native Hawaiian/Pacific Islander, *M* million

^a^Electronic Residency Application Service (ERAS) data include other race/ethnicity and unknown race/ethnicity, and do not have a multi-race designation

^b^Includes diagnostic radiology and interventional-radiology-integrated residents with International Medical Graduate (IMG) pathway, U.S. and Canadian medical graduates and U.S. doctor of osteopathic medicine graduates

^c^Number of full-time faculty at all U.S. medical schools as of Dec. 31, 2020

By the 2020 U.S. Census, women comprised 50.8% (168,602,022) of the total population [10]. Although they represented 49.5% of medical school graduates, women who were diagnostic radiology residency applicants (27.5%), diagnostic radiology residents (35.9%), diagnostic radiologists (26.5%) and diagnostic radiology faculty members (29.9%) were all significantly underrepresented within the specialty [7–9, 11] (Table 1).

## Radiology residency applicants

**Fig. 1:**
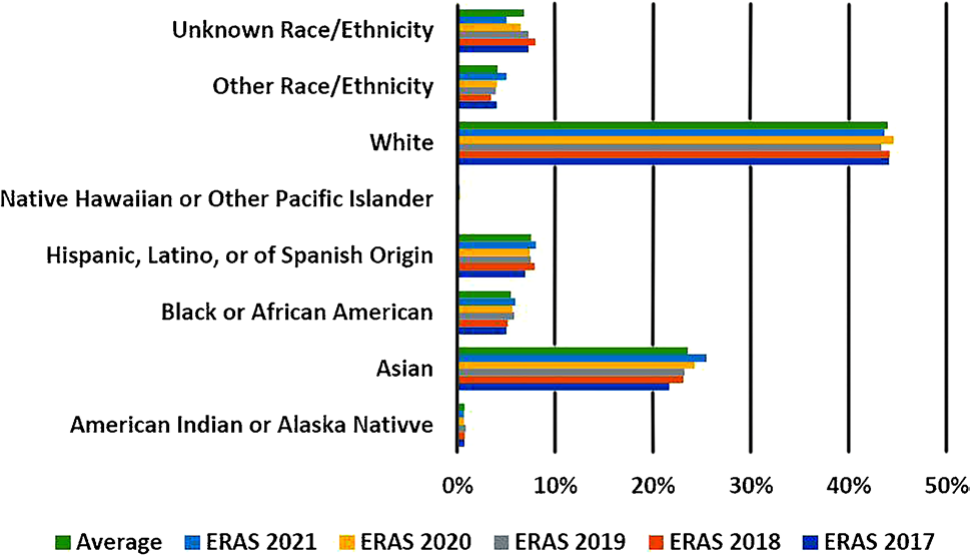
Diagnostic radiology data. The percentage of applicants according to individual race and ethnicity as a portion of the total applicant pool for diagnostic radiology residency programs. The graph shows no significant change in the most recent 5-year period (2017–2021) of Electronic Residency Application Service (ERAS) data

In the 5-year period from 2017 to 2021, the number of applicants to diagnostic radiology programs averaged just under 2,400, with a low of 2,274 (2019) and a peak of 2,577 (2021) [8]. Although there was a gradual increase in the number of URiM applicants to radiology residency programs over this time period, their percentage of the total applicant pool by individual racial and ethnic groups did not significantly change. This is also true for the two largest groups, Whites and Asians, whose percentage of the total applicant pool by racial and ethnic grouping also did not significantly change (Fig. 1).

**Fig. 2:**
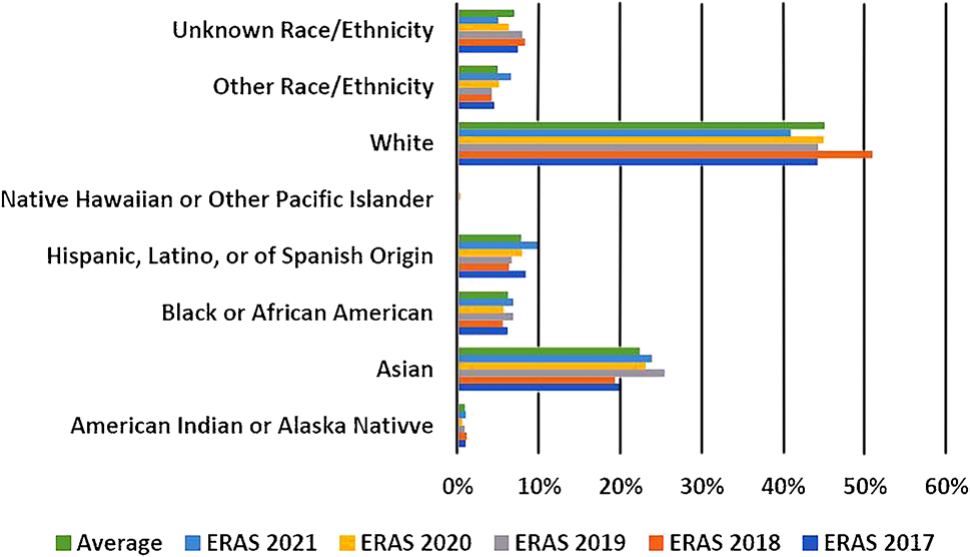
Interventional radiology data. The percentage of applicants according to individual race and ethnicity as a portion of the total applicant pool for interventional radiology (IR)-integrated programs. The graph shows no significant change in the most recent 5-year period (2017–2021) of Electronic Residency Application Service (ERAS) data

Over the last 5 years the number of applicants to interventional radiology (IR)-integrated residency programs has averaged just under 790, with a low of 452 (2019) and a peak high of 1,080 in 2021 [8]. Although there has been a gradual increase in the number of URiM applicants to IR-integrated residency programs over time, their percentage of the total applicant pool by individual racial and ethnic groups has not significantly changed (averaging 6.8% for Blacks/African Americans and 7.8% for Hispanics/Latinos/Spanish origin applicants) [8]. Among White and Asian applicants, their percentage of the total applicant pool by racial and ethnic grouping also has not significantly changed [8] (Fig. 2).

**Fig. 3:**
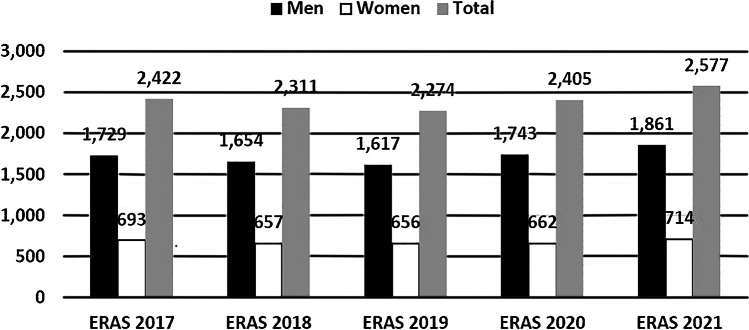
Data by gender. In the most recent 5-year period (2017–2021) of Electronic Residency Application Service (ERAS) data, the number of men and women applicants to diagnostic radiology residencies as a proportion of the whole applicant pool has not significantly changed

The average number of applicants who self-identified as American Indian or Alaska Native during the 5-year period is 18, against a background of the total number of applicants in the same period averaging just under 2,400 [8]. For Native Hawaiian or Pacific Islander populations, the average number of applicants during the same time period was three per year [8]. In this same 5-year period, the Electronic Residency Application Service (ERAS) reported that the percentage of women in the application pool for diagnostic radiology residency programs has remained relatively unchanged, peaking at 28.8% in 2019 with a drop to a low of 27.5% in 2020 [8] (Fig. 3).

## Diagnostic radiology practicing physicians and faculty

In the 10-year period between the 2010 and 2020 U.S. censuses, the number of radiologists in practice increased by 1,954 (from 26,054 to 28,008), representing a 7.5% increase [9]. For radiologists who self-identified as URiM, there was a net increase of 0.5%, or an additional 114 radiologists in practice, representing 2.6% of practicing radiology physicians [9]. For women there was a 3% increase, or an additional 1,278 practicing female radiologists. Women comprised slightly more than one-third (34.5%) of the increase in practicing diagnostic radiologists [12].

During the same period there was a 6-fold increase in the number of radiology faculty members, from 1,408 to 10,213 [7, 9]. While there was an increase in the number of faculty positions held by radiologists who self-identified as URiMs (from 28 to 228), there was a net decrease of 1.5% of the total radiology faculty positions held by URiM radiologists. This was largely the result of a decrease in the percentage of the total number of faculty members who self-described as Hispanic or Latino, or of Spanish origin. Women accounted for 30% of the increase in diagnostic radiology faculty, with an additional 2,684 positions filled in the 10-year period [7, 12].

The next largest increase in faculty positions occurred for those who self-identified as Asian, with a 15.1% increase and an additional 2,588 faculty positions. For those who identified as Asian alone or in combination, the percentage of total applicants (24.4% to 25.6%), diagnostic radiology residents (26.2% to 27.8%), practicing physicians (13.4% to 16.9%) and academic radiologists (9.2% to 28.5%) all increased [7–9].

## The problem we all live with

“The Problem We All Live With” is the title of a portrait by Norman Rockwell depicting 6-year-old Ruby Bridges (Hall) being escorted by four federal marshals to her first day of class at the public all-White William Frantz Elementary School in New Orleans on Nov. 14, 1960, 6 years after the Supreme Court landmark decision Brown versus Board of Education.

When Bridges was in kindergarten, she was one of many Black students in New Orleans chosen to take a test determining whether she could attend a White school. It is said the test was written to be especially difficult so that students would have a hard time passing. The idea was that if all the Black children failed the test, New Orleans schools might be able to stay segregated for a while longer. The decision to integrate New Orleans public schools was challenged by the State Legislature throughout the summer and early fall until the Legislature ran out of stalling tactics.

Ruby Bridges’ story of forced school integration draws interesting parallels to our current diversity dilemma. Her story might offer some insights on why there is increasing racial and ethnic population diversity in U.S. Census numbers but only modest gains in diversifying the physician health care workforce for radiology. With population growth in the 10-year period between the two most recent U.S. censuses there have been increases in racial and ethnic groups, as described, but there remains persistent underrepresentation among medical school graduates, diagnostic radiology applicants, residents, practicing physicians and medical school faculty members.

In exploring the “why” question, several factors should be given consideration, including the imposed limitations on the size of the available pool of medical school matriculants and graduates from URiM groups, exposure to the specialty, selection criteria (at both undergraduate and postgraduate levels), outreach and mentorship.

However, one cannot ignore, or underestimate, the contributions of two insidious behaviors — unconscious (implicit) bias and amplification cascade — that serve to undermine our best efforts to increase diversity of the radiology health care workforce [13].

Researchers have documented unconscious bias in a variety of contexts and professions, including health care, in which they have studied differential treatment, diagnosis, prescribed care, patient well-being and compliance, physician–patient interactions, clinical decision-making and medical school education [14, 15]. Unconscious bias (implicit bias) is bias that results from the tendency to process information based on unconscious associations and feelings, even when these are contrary to one’s conscious or declared beliefs. The publication *The HR Source* identified five types of unconscious bias: affinity bias, halo effect, horns effect, attribution bias and confirmation bias [16].

Affinity bias leads us to favor people who we feel we have a connection or similarity to. Halo effect occurs when we perceive one great thing about a person and let the glow of that one thing color our opinions of everything else about that person. Horns effect occurs when one’s perception of someone is unduly influenced by one negative trait. Attribution bias occurs when, in our assessment of others, we are more likely to consider the achievements of others as a result of luck or chance and their failings as a result of their personality or behavior. Confirmation bias is the tendency to search for, interpret, focus on and remember information that aligns with our preconceived opinions. Each of these forms of bias can individually or collectively affect our decision-making about a candidate in the admissions process and in interviews for residency training, jobs or academic appointments.

In the September 2018 issue of *Academic Medicine*, Teherani and colleagues [13] at the University of California San Francisco School of Medicine identified differences in grading that consistently favored non-URiM students. In their analysis, URiM students received half as many honors grades as non-URiM students and were three times less likely to be selected for honor society memberships. The authors identified the phenomenon of amplification cascade, in which small differences in assessed performance lead to large differences in grades and selection for awards [13]. Ultimately, amplification cascade can impact the residency selection process and subsequent recruitment for faculty positions, with significant consequences that can affect careers.

## Strategies to help promote diversity

The data here demonstrate that a major impediment to increasing diversity in medicine, and our specialty, is the pipeline of URiM candidates who are admitted to our medical schools. In recent years, the number of women admitted and graduating from U.S. medical schools has reached near parity with men, although women still remain underrepresented among diagnostic radiology applicants, residents, practicing physicians and faculty [9, 17].

One very important component of increasing the URiM health care workforce lies within the corridors of our medical schools and, specifically, the admissions policies that effectively limit opportunities for students who might have overcome significant obstacles — under-resourced educational programs, lack of finances or social capital for career-building — to achieve a level of success that allows them to even consider careers in medicine. A more holistic approach to medical school admissions and successful matriculation would value the road traveled and offer student support services that address structural barriers within institutions that limit success of underrepresented students [18, 19].

Untapped resources could provide a ready supply of qualified candidates for admission. This would require expanding outreach to programs that have not traditionally been considered by academic medicine. There are 107 historically black colleges and universities (HBCUs) with enrollment of 228,000 students. If only 10% of these students were pursuing premedical education, that number would be almost half the number of first-time applicants (46,758) to medical school in 2021–2022 [20]. Targeting these institutions prior to medical school matriculation with opportunities to engage in mentorship and research within our specialty might provide avenues for increasing diversity in radiology [19, 21].

The American College of Radiology’s Pipeline Initiative for the Enrichment of Radiology (PIER) program offers PIER internships to first-year medical students at institutions throughout the United States [17]. The PIER program provides opportunities for URiMs and women to explore the radiology specialty and engage in research. Accepted scholars are paired with at least one preceptor in the student’s area of interest. The program underscores the importance of outreach and the role that individuals and departments can play in increasing diversity in the specialty.

Strategies can be employed by individuals and organizations to mitigate the effects of unconscious (implicit) bias. For individuals, it is important to acknowledge that unconscious bias exists in all of us. The defense against it requires a commitment to educating oneself about how bias can affect one’s behavior. It requires adapting strategies that counterbalance our biases, such as self-reflection, with questions that challenge our preconceived notions/opinions. Individuals might want to learn more about their bias tendencies [14]. At Project Implicit, people can take an Implicit Association Test on a variety of topics including race, age, sex, gender, weight, skin tone, etc., to inform themselves about their unconscious biases [22]. Individuals responsible for interviewing, selection and hiring within their department should be encouraged to participate in bias training sessions [18]. Interview teams should be composed of diverse members to make more transparent the operation of biases [14].

Within radiology departments, it is important for leaders to promote the development and implementation of strategies that support diversity as an institutional policy and cultivate a shared responsibility among members to create inclusive learning and working environments. Leaders should make available unconscious bias training sessions for all residency and faculty interviewers, and for anyone in the organization who wants to be more aware of the effects of bias on their behavior with colleagues, trainees, staff and patients. It is equally important that diversity and inclusion be integrated into the core mission with a review of policies, practices and programs, and that change be instituted as needed to achieve diversity priorities [14, 23].

Departments should provide opportunities and resources for professional development to empower and equip members to accomplish diversity-related goals [14]. This could take the form of a line item in the department budget, similar to line items for education and research programs. The department should evaluate programs by race, ethnicity and gender to look for patterns of recruitment, retention, mentoring programs, research opportunities and appraisal processes that might be affected by unconscious bias.

At the residency selection level, it is important to acknowledge that the existence of bias in the selection process might be increasing the effects of application cascade [13]. Residency program directors need to consider a more holistic approach to the selection process that acknowledges the fact that our educational environments are not colorblind, nor are they equitable for URiM students [18, 19, 21].

## Conclusion

The 2020 U.S. Census demonstrated that the demographics of the country are changing toward increasing population diversity. This shift provides medicine and radiology the opportunity to respond to the challenge of expanding the diversity of their physician workforce and improve health equity. Strategies directed at expanding the pipeline for medicine that are aimed at the enhancement of gender, racial and ethnic diversity among students who might apply to medical school can help to increase the available pool of diverse candidates for medicine in general and radiology more specifically.
