# Correction to: Diversity in radiology: the right thing to do, the smart thing to do

**DOI:** 10.1007/s00247-022-05507-3

**Published:** 2022-09-09

**Authors:** Theodore R. Hall, Kathleen Brown

**Affiliations:** 1 UCLA Department of Radiology, Section of Pediatric Imaging, 757 Westwood Plaza, Los Angeles, CA 90095 USA; 2 UCLA Department of Radiology, Section of Cardiothoracic Imaging, Los Angeles, CA USA

**Correction to: Pediatric Radiology (2022) 52:1711–1718**

10.1007/s00247-022-05416-5

In the last paragraph of the section entitled “Strategies to help promote diversity” (page 1717), the first sentence has been corrected to read:

At the residency selection level, it is important to acknowledge that the existence of bias in the selection process might be increasing the effects of amplification cascade [13].

The word “application” has been corrected to “amplification.”

