# Correction to: Diversity in radiology: the right thing to do, the smart thing to do

**DOI:** 10.1007/s00247-023-05661-2

**Published:** 2023-04-13

**Authors:** Theodore R. Hall, Kathleen Brown

**Affiliations:** 1 UCLA Department of Radiology, Section of Pediatric Imaging, 757 Westwood Plaza, Los Angeles, CA 90095 USA; 2 UCLA Department of Radiology, Section of Cardiothoracic Imaging, Los Angeles, CA USA

**Correction to: Pediatric Radiology (2022) 52:1711–1718**

**https://doi.org/10.1007/s00247-022-05416-5**

A corrected version of Table 1 is provided. The following corrections have been made:

- “Diagnostic Radiology Residents” Men – 3,459 (73.5)
- “Diagnostic Radiology Residents” Women – 1,244 (26.5)
- “Diagnostic Radiology Residents” Total Non-duplicated – 4,703
- “U.S. Medical Graduates” Total Non-duplicated – 20,390
- “Diagnostic Radiology Applicants” Total Non-duplicated – 2,405

**Table 1:** 2020 demographic distribution by gender, Hispanic ethnicity and race of the U.S. population, U.S. medical school graduates, diagnostic radiology applicants, residents, practicing physicians and faculty members

	Gender		Ethnicity		Race					Total Non-duplicated
Group	Men	Women	Hispanic	Non-Hispanic	White	Asian	Black	AI/AN/NH/PI	Multi-race	
2020 U.S.Census	163.2 M (49.2)	168.6 M (50.8)	**60.7 M (18.5)**	197.2 M (60.1)	250.4 M (76.3)	19.4 M (5.9)	**44.0 M (13.4)**	**4.9 M (1.5)**	9.2 M (2.8)	328,239,523
U.S.medical school graduates	10,280 (50.4)	10,110 (49.5)	*1,063 (5.3)*	504 (2.5)	**10,897 (54.6)**	4,299 (21.6)	*1,238 (6.2)*	*47 (0.3)*	1,598 (8.0)	20,390
Diagnostic radiology applicants^a^	1,743 (72.5)	662* (27.5)*	**171 (8.1)**	254 (12.1)	**986 (47.1)**	528 (25.2)	**132 (6.3)**	21 (1.0)		2,405
Diagnostic radiology residents^b^	3,459* (73.5)*	**1,244 (26.5)**	**302 (6.9)**	222 (5.0)	*2,583 (59.2)*	1,190 (27.8)	**173 (3.9)**	*8 (0.2)*	187 (4.3)	4,703
Diagnostic radiology practicing physicians	20,595 (73.5)	**7,413 (26.5)**	**1,106 (4.4)**	480 (1.9)	18,252 (73.0)	4,240 (16.9)	**658 (2.6)**	**75 (0.3)**	187 (0.7)	**28,008**
Diagnostic radiology faculty^c^	7,161 (70.1)	**3,052 (29.9)**	*239 (2.5)*	216 (2.2)	*5,915 (62.0)*	2,718 (28.5)	**228 (2.3)**	**21 (0.2)**	198 (2.0)	10,213

Based on comparison of the 2010 and 2020 U.S. censuses, numbers in **bold** represent increases, *italics* represent declines; percentages for both are in parentheses for the U.S. population, U.S. medical school graduates, radiology applicants, residents, practicing physicians and radiology faculty members (Comparison of data abstracted from [5])

*AI/AN/NH/PI* American Indian/Alaskan native/Native Hawaiian/Pacific Islander, *M* million

^a^ Electronic Residency Application Service (ERAS) data include other race/ethnicity and unknown race/ethnicity, and do not have a multi-race designation

^b^ Includes diagnostic radiology and interventional-radiology-integrated residents with International Medical Graduate (IMG) pathway, U.S. and Canadian medical graduates and U.S. doctor of osteopathic medicine graduates

^c^ Number of full-time faculty at all U.S. medical schools as of Dec. 31, 2020

Of note, a comparison of this 2020 data with the 2010 data published by Chapman et al. [5] shows that compared to the 2010 demographic data (below) there was no significant change in the number or percentage of men and women in DR and IR residency training programs:

- Men – 3,271 (72.2)
- Women – 1,260 (27.8)
- Total Non-duplicated – 4,531

On page 1714 within the paragraph beginning with “By the 2020 U.S. Census. Women…”, the correct percentage for the women who were diagnostic radiology residents is 26.5% (instead of 35.9%). The point being made about underrepresentation remains valid.

